# Microalgae (*Chlorella vulgaris*) attenuates aflatoxin-associated renal injury

**DOI:** 10.3389/fphar.2023.1291965

**Published:** 2023-12-27

**Authors:** Ahmed Abdeen, Rasha Elsabagh, Sawsan S. Elbasuni, Alshaimaa M. Said, Afaf Abdelkader, Ali H. El-Far, Samah F. Ibrahim, Ostan Mihaela, Liana Fericean, Abdelfattah M. Abdelfattah, Mohamed El-Hewaity, Nady Elbarbary, Amgad Y. Kadah, Samar S. Ibrahim

**Affiliations:** ^1^ Department of Forensic Medicine and Toxicology, Faculty of Veterinary Medicine, Benha University, Toukh, Egypt; ^2^ Department of Food Hygiene and Control, Faculty of Veterinary Medicine, Benha University, Toukh, Egypt; ^3^ Department of Avian and Rabbit Diseases, Faculty of Veterinary Medicine, Benha University, Toukh, Egypt; ^4^ Department of Biochemistry, Faculty of Veterinary Medicine, Benha University, Toukh, Egypt; ^5^ Department of Forensic Medicine and Clinical Toxicology, Faculty of Medicine, Benha University, Benha, Egypt; ^6^ Department of Biochemistry, Faculty of Veterinary Medicine, Damanhour University, Damanhour, Egypt; ^7^ Department of Clinical Sciences, College of Medicine, Princess Nourah bint Abdulrahman University, Riyadh, Saudi Arabia; ^8^ Department of Biology and Plant Protection, Faculty of Agriculture. University of Life Sciences “King Michael I" from Timișoara, Timișoara, Romania; ^9^ Department of Clinical Pathology, Faculty of Veterinary Medicine, University of Sadat City, Sadat City, Egypt; ^10^ Department of Pharmacology, Faculty of Veterinary Medicine, Menoufia University, Shebin Elkoum, Egypt; ^11^ Department of Food Hygiene, Faculty of Veterinary Medicine, Aswan University, Aswan, Egypt; ^12^ Department of Physiology, Faculty of Veterinary Medicine, Benha University, Toukh, Egypt; ^13^ Department of Zoology, College of Science, King Saud University, Riyadh, Saudi Arabia

**Keywords:** oxidative stress, inflammatory cytokines, apoptosis, residues, Japanese quail, computational modeling

## Abstract

**Introduction:** Aflatoxins (AFT) are ubiquitous environmental pollutants that are extremely dangerous for both human beings as well as animals. A safe, effective, and considerate strategy is therefore credited with controlling AFT intoxication. Therefore, our study aimed to evaluate the mitigating properties of *Chlorella vulgaris* (ChV) against AFT-induced nephrotoxicity and altered egg quality.

**Methods:** Quails were randomized into Control group (receiving a normal diet); ChV group (1 g/kg diet); AFT group (receiving an AFT-containing diet); and the AFT-ChV group were given both treatments.

**Results and discussion:** AFT provoked kidney injury, exhibited by increased renal biochemical parameters and reduced protein levels. Malondialdehyde (MDA) levels dramatically increased as a consequence of AFT exposure, and glutathione (GSH) levels, superoxide dismutase (SOD), and glutathione peroxidase (GPx) activities were also decreased. Substantial up-modulation of the mRNA expression of the inflammatory cytokines (TNF-α, IL-1β, and IL-6) was additionally reported. Furthermore, AFT residues were detected in the egg compromising its quality and nutritional value. Contrarily, ChV supplemented diet suppressed the AFT-prompted oxidative stress and inflammation, together with enhancing the nutritional value and quality of eggs and decreasing AFT residues. These beneficial impacts are proposed to be attributed to its antioxidant and nutritional ingredients. The molecular docking dynamics confirmed the inflammatory and apoptotic protein targets for ChV. Our findings recommend that adding ChV supplements to foods might guard against nephrotoxicity brought on by AFT exposure.

## 1 Introduction

Aflatoxins (AFTs), di-furanocoumarin metabolites, accumulate in food and fodders due to contamination with *Aspergillus flavus* and *Aspergillus parasiticus* strains (Zhang et al., 2022). The global climatic shift caused by global warming offers favorable conditions for fungi to grow and generate AFTs in contaminated crops, which can build up to lethal concentrations (Medina et al., 2017). The Food and Drug Administration (FDA) has classified AFTs as ineluctable contaminants due to their pervasiveness along with their potential jeopardy to the health of humans and animals (Abdel-Daim et al., 2021). AFT intoxication has genotoxic, mutagenic, and carcinogenic impacts and is designated by the International Agency for Research as a class-I carcinogen in various organs (Hebels et al., 2009; Ahmed et al., 2022).

The toxicity of AFTs is principally due to their metabolism to a more toxic metabolite, namely, aflatoxin-exo-8,9-epoxide (AFTO) through hepatic cytochrome-P450 biotransformation (Zhu et al., 2020; Yilmaz and Bag, 2022). AFTO is an extremely reactive intermediate that targets DNA’s guanine residues, resulting in DNA adducts and mutations. Thereafter, AFTO is detoxified by the phase-II glutathione (GSH) system to a minimally toxic GSH complex, which is then excreted from the cells via the mercapturic acid pathway (Dlamini et al., 2021). It is intriguing that in addition to the liver (Ahmed et al., 2022), the kidney is a probable target since AFT and AFTO are preferentially absorbed and concentrated by tubular cells, and their accumulation in the renal medulla before being expelled in the urine causes direct renal tubular damage (Wang et al., 2022).

There is ample proof indicating the embroilment of oxidative stress, inflammatory responses, and apoptotic pathway in AFT-induced nephrotoxicity (Owumi et al., 2020; Wang et al., 2022). Thus, excess generation of reactive oxygen species (ROS), with consumption of antioxidant enzymes after AFT intoxication, provokes tissue injuries such as DNA mutations, mitochondrial disruption, improper folding of protein, and ultimately necroptosis (Dlamini et al., 2021; Elgazzar et al., 2022; Li et al., 2022). Moreover, the stability of AFTs and their metabolites highlights the necessity for developing secure, feasible, and efficient strategies for reducing their damaging impacts (Kolosova and Stroka, 2012). Therefore, supplementation with natural antioxidants could be an appropriate therapeutic approach to counteract AFT-induced kidney damage.

*Chlorella vulgaris* [Chlorellaceae; *Chlorella vulgaris* var. vulgaris], ChV, the most renowned microalgae that pertain to the Chlorophyta class, has outstanding nutritional value, owing to their enrichment in bioactive compounds such as long-chain polyunsaturated fatty acids (PUFAs), phenolic compounds, proteins, amino acids, peptides, and vitamins (Andrade et al., 2018; Elbasuni et al., 2022), which play a beneficial role in animal and human health (Chen et al., 2023). Plenty of studies have been published about ChV as a splendid natural antioxidant, anti-inflammatory, and antiapoptotic agent (Abdelnour et al., 2019b; Abdelhamid et al., 2020). Accordingly, Accumulating literature has emphasized the effectiveness of ChV against a variety of nephrotoxic agents, including, mercury chloride (Blas-Valdivia et al., 2011), cadmium (Shim et al., 2009), and gentamicin (Al-Halaseh et al., 2022).

In accordance with this assertion, we speculated that ChV supplementation might mitigate oxidative stress and inflammation induced AFT. Consequently, the present study was intended to evaluate the modulatory impact of ChV as a feed supplement against AFT bioaccumulation and nephrotoxicity. Biochemical and oxidative stress markers, as well as pro-inflammatory-related gene expression, were evaluated in this study. In addition, quail production and egg quality were evaluated.

## 2 Materials and methods

### 2.1 Experimental approach

Japanese quails (*Coturnix japonica*; 10 weeks old with an average body weight of 200–250 g) were bought from the Faculty of Veterinary Medicine at Benha University in Egypt. Quails received a commercial maize and soybean meal baseline diet that satisfied all their nutritional needs according to the guidelines set forth by the NRC (1994) and free access to water as needed during the trial. They were reared in a well-ventilated room (25°C ± 2°C, humidity 55%–60%, and 17 h of light/day). Quails were allotted randomly over the deep litter floor pen compartments each consisting of eight females and two males. Following 2 weeks of acclimation, the quails were randomized and assigned into four groups, each group consisting of three replicates of five birds: the control group—fed on a basal diet; the ChV group—fed on a basal diet supplemented with a dried powder of whole ChV (1 g/kg) (Marine Toxins Lab, National Research Centre, Egypt) (Abdelnour et al., 2019b); the AFT group—fed on an AFT-contaminated diet (50 ppb; AFT mix, purity >98%, Merck, Darmstadt, Germany) as described by Elbasuni et al. (2022); and the AF + ChV group—fed on an AFT-contaminated diet along with ChV for 3 weeks. The same management, sanitary, and environmental standards were followed in the rearing of birds in each experimental group.

### 2.2 Productive performance assessment

Eggs were gathered and checked for deformity to calculate the proportion of cracked eggs. Egg mass was calculated by multiplying the number of eggs by their average weight.

### 2.3 Evaluation of egg nutritional values

Randomly selected eggs from each group were gathered, homogenized, and stored at 80°C for later analysis. Egg protein, fat, cholesterol, and triacylglycerol levels were assessed spectrophotometrically.

### 2.4 Biochemical analyses

Blood samples were drawn from the jugular vein, and sera were harvested and stored at −20°C for further biochemical analysis [urea (Cat# UR2110), creatinine (Cat# CR1250), uric acid (Cat# UA2120), total protein (Cat# TP2020), and albumin (Cat# AB1010) levels] at the end of the trial. All methods were conducted following the manufacturers’ instructions (Biodiagnostic, Cairo, Egypt).

### 2.5 Determination of renal tissue LPO and antioxidant enzyme activities

At the end of the trial, the quails were euthanized in the absence of other birds to reduce the distress. The birds were first anesthetized using 2% isoflurane inhalation followed by cervical dislocation. Kidney specimens from the humanely sacrificed quails were obtained and stored at −80°C until evaluation of tissue oxidation indices. Renal tissues were discretely homogenized in phosphate buffer, pH 7.4, and the homogenate was used to determine the MDA (Cat# MD2529) and GSH (Cat# GR2511) levels, as well as the SOD (Cat# SD2521) and GPx (Cat# GP2524) activities, according to the manufacturer’s recommendations (Biodiagnostic).

### 2.6 Quantitative real-time PCR

Using the RNeasy Mini Kit (Cat# 74104, QIAGEN Sciences Inc., Germantown, MD, United States), the total RNA was extracted in accordance with the manufacturer’s instructions. The primer sequences for the targeted genes (housekeeping gene 28S rRNA and the inflammatory cytokines TNF-α, IL-1β, and IL-6) are presented in Supplementary Table S1. The QuantiTect probe RT-PCR kit (Cat#204443, QIAGEN Sciences Inc.) was employed to carry out quantitative real-time PCR (qRT-PCR). Real-time PCR equipment (Applied Biosystems, Waltham, CA, United States) was used to capture signals. The cycling conditions for PCR were as follows: 50°C for 30 min, 94°C for 10 min, 40 cycles at 94°C for 15 s, and 60°C for 1 min. The cycle threshold (Ct) values and amplification curves were calculated using Stratagene Mx3005P software. Following normalization using the housekeeping gene, the 2Ct technique was used to calculate the fold changes in the expression levels, as described in Habotta et al. (2023).

### 2.7 Detection of aflatoxin residues in eggs

The egg was collected after the completion of the trial to detect the residual AFT using high-performance liquid chromatography (HPLC). Aflatoxin M1 reference materials (Sigma-Aldrich, St. Louis, MO, United States) were used in the analyses.

### 2.8 Molecular docking assessment

The three-dimensional structures of *Coturnix japonica*’s extracellular SOD1, SOD2, GPx, GR, GCLC, GSH synthase, IL1RAP, IL6RA, TNFRSF1A, and TRAF1 were generated using the Robetta server (Baek et al., 2021). Proteins were prepared for docking using Molecular Operating Environment (MOE 2015.10, Chemical Computing Group, Montreal, QC, Canada) software. The three-dimensional structures of AFB1, AFB2, AFG1, and AFG2 were retrieved from the PubChem (https://pubchem.ncbi.nlm.nih.gov/) database. In addition, bioactive compounds of ChV were retrieved from PubChem and LOTUS: Natural Products Online (https://lotus.naturalproducts.net/) databases. Furthermore, MOE software was used for molecular docking, protein–ligand interaction analysis, and visualization.

### 2.9 Statistical analyses

One-way analysis of variance (ANOVA) with the LSD *post hoc* test was used to analyze the collected data. Prior to performing ANOVA, all data were tested for normality (Shapiro–Wilk and Kolmogorov–Smirnov tests; *p* > 0.05) and homogeneity of variances (Levene’s test; *p* > 0.05). All analyses were performed using SPSS 25 software for Windows (SPSS Inc., Chicago, IL, United States). Data were represented as the mean ± SE. The data were considered statistically significant at *p*-values <0.05. Additionally, RStudio’s R version 4.0.2 was used to create a debiased sparse partial correlation (DSPC) algorithm network, clustering heatmap, and variable importance projection (VIP) score.

## 3 Results

### 3.1 Egg productive performance

The effect of ChV supplement on the productivity of quails that received a diet contaminated with AFT is shown in Table 1. The egg number, weight, and mass decreased significantly, while cracked eggs increased dramatically in AFT-intoxicated birds versus all other treated groups. With daily observation, the birds that fed the AFT diet showed watery droppings, abnormal gait, and ruffled feathers. On the other hand, the co-exposure to AFT and ChV caused marked betterment of the production performance of birds. Intriguingly, the ChV group surpassed the control group in terms of egg number, weight, and mass.

**TABLE 1:** Egg productive performance parameters of laying Japanese quails following ChV supplementation and/or AFT exposure.

Parameter	Experimental group			
	Control	ChV	AFT	AFT–ChV
Egg no./bird	20.45 ± 1.16	22.21 ± 0.9 *	17.15 ± 0.62*	20.76 ± 0.31 #
Egg weight (g)	12.09 ± 0.01	13.41 ± 0.22 *	11.01 ± 0.11*	12.49 ± 0.04 *#
Egg mass (g)	247.18 ± 14.03	297.63 ± 10.58 *	188.94 ± 8.68*	259.4 ± 4.53 #
Cracked egg %	0.21 ± 0.21	00.00 ± 00.00	8.13 ± 1.02*	1.39 ± 0.42 #

AFTs, aflatoxins; ChV, *Chlorella vulgaris*. Values are represented as mean ± SE. *p < 0.05 vs. control group; #*p* < 0.05 vs. AFT group.

### 3.2 Changes in the egg nutritional value following exposure to ChV and/or AFT

Table 2 shows that compared to controls, the ChV group showed a significant increase in the egg protein content while a significant decrease in egg fat, cholesterol, and triacylglycerol levels. ChV supplementation to quails that fed on an AFT-contaminated diet resulted in a noticeable increase in egg protein and a decrease in fat and cholesterol contents compared to the AFT group.

**TABLE 2:** Effect of ChV supplement on egg nutritive value of laying Japanese quails that fed on an AFT-contaminated diet.

Parameter	Experimental group			
	Control	ChV	AFT	AFT–ChV
Protein (%/g)	15.39 ± 0.38	18.29 ± 0.44 *	12.10 ± 0.29 *	14.26 ± 0.31 #
Fat (%/g)	1.23 ± 0.03	0.57 ± 0.02 *	1.96 ± 0.05 *	1.72 ± 0.04 *#
Cholesterol (mg/g)	142.92 ± 3.54	128.56 ± 1.79 *	176.84 ± 4.22 *	161.76 ± 3.74 *#
Triacylglycerol (mg/100 g)	87.74 ± 2.17	64.69 ± 1.73 *	102.42 ± 2.45 *	96.13 ± 2.07 *

AFTs, aflatoxins; ChV; *Chlorella vulgaris*. Values are represented as mean ± SE. *p < 0.05 vs. control group; #*p* < 0.05 vs. AFT group.

### 3.3 Serum biochemical indices

**FIGURE 1:**
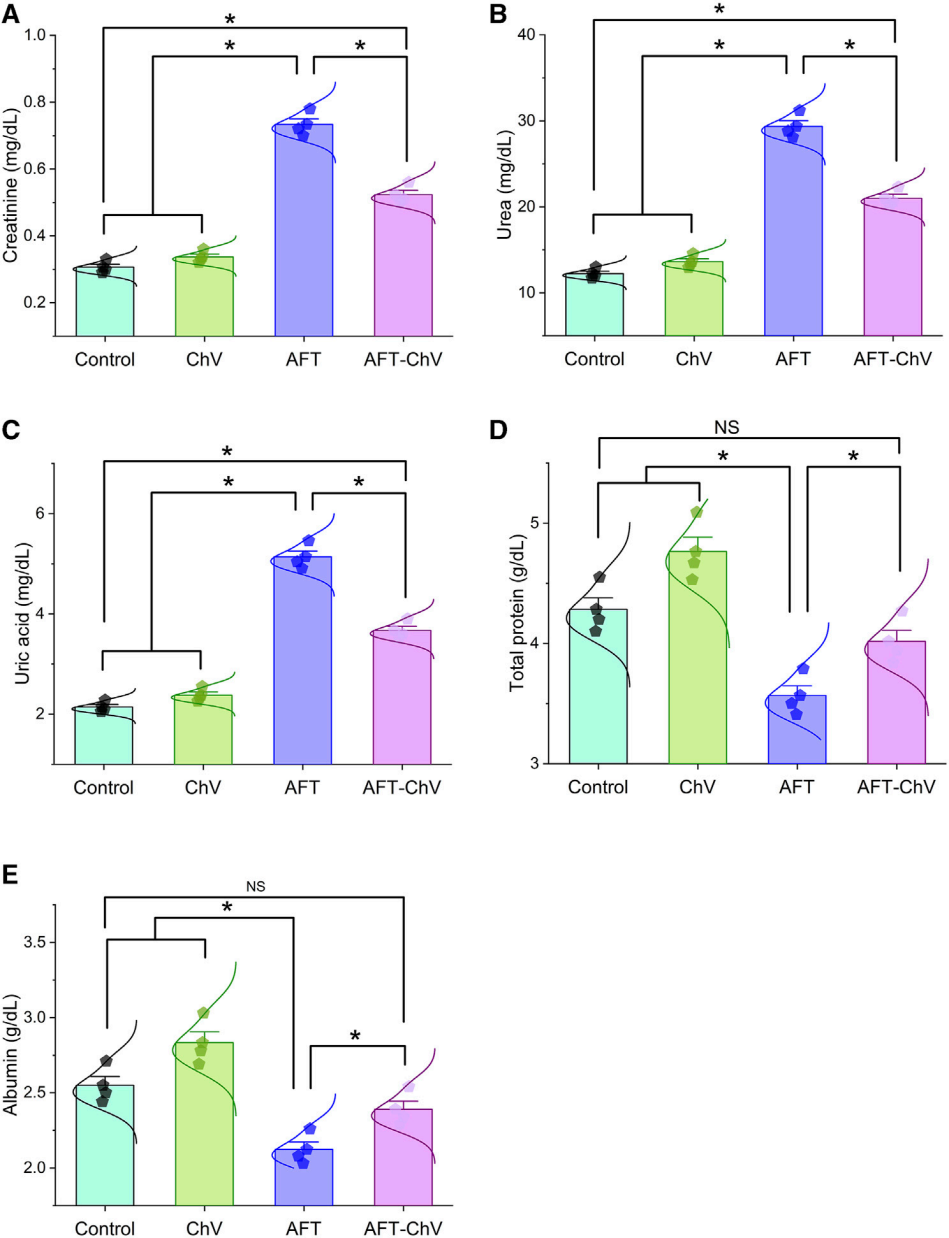
Bar-dot plot panel of serum biochemical tests upon ChV and/or AFT exposure. **(A)** Creatinine, **(B)** Urea, **(C)** Uric acid, **(D)** Total protein, and **(E)** Albumin. AFT, aflatoxins; ChV, *Chlorella vulgaris*. Values are represented as mean ± SE (*p < 0.05).

In accordance with the findings depicted in Figure 1, quails that fed on an AFT-contaminated diet demonstrated a substantial increase in the kidney function test, including creatinine, urea, and uric acid, alongside a decrease in total protein and albumin levels, when confronted with those fed on a basal diet. In contrast, concurrent consumption of a diet containing ChV and AFT demonstrated amelioration in the renal function test and protein levels compared to those exposed to AFT solely.

### 3.4 Lipid peroxidation and antioxidant parameter assay

**FIGURE 2:**
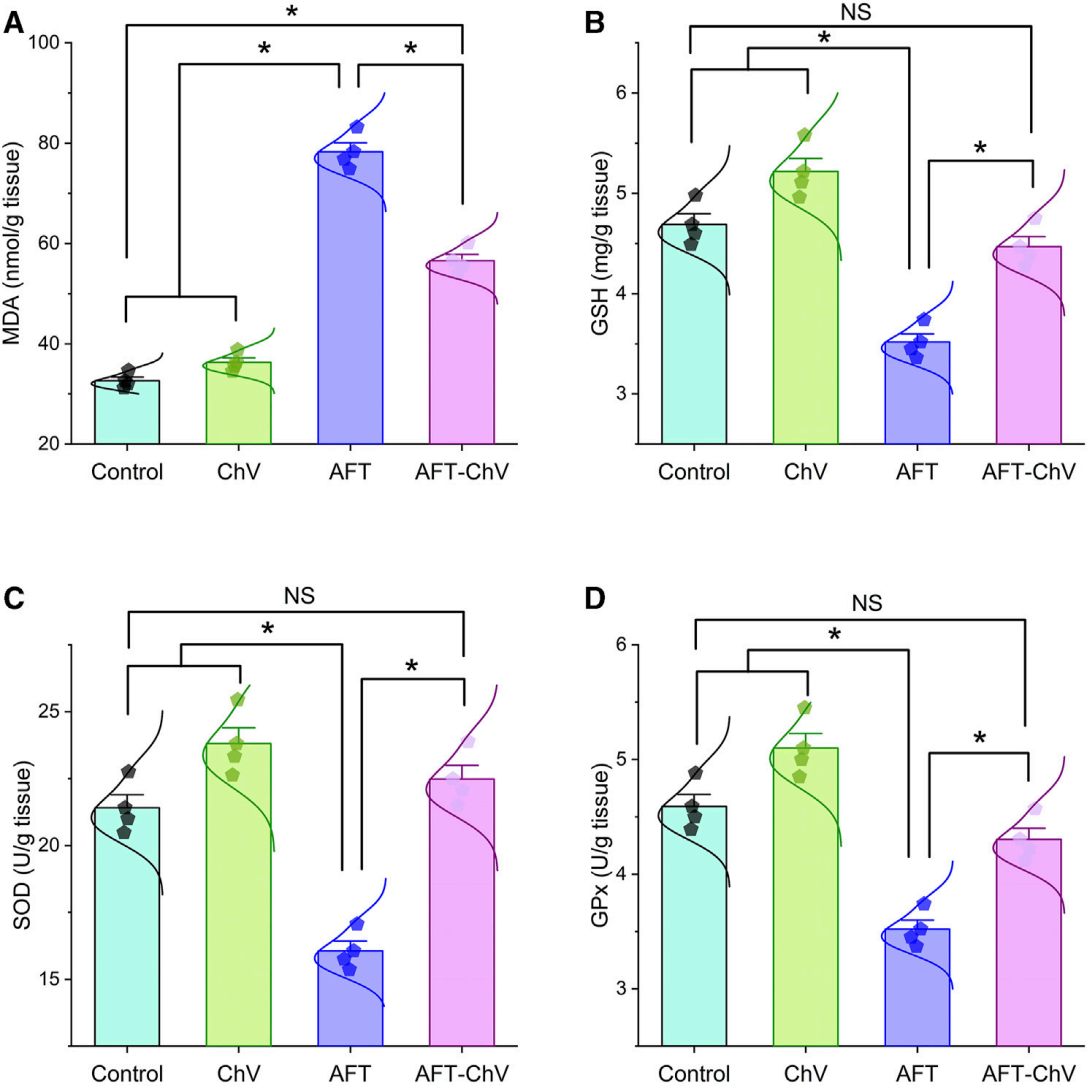
Bar-dot plot panel of oxidant/antioxidant indices following ChV and/or AFT treatment in the kidney tissue. **(A)** MDA, **(B)** GSH, **(C)** SOD, and **(D)** GPx. AFTs, aflatoxins; ChV, *Chlorella vulgaris*; GPx, glutathione peroxidase; GSH, reduced glutathione; MDA, malondialdehyde; SOD, superoxide dismutase. Values are represented as mean ± SE (*p < 0.05).

AFT induced an obvious state of oxidative damage and lipid peroxidation, as shown in Figure 2. A substantial increase in the renal malondialdehyde (MDA) level together with a discernible reduction in the reduced-glutathione (GSH) level and the enzyme activities of superoxide dismutase (SOD) and glutathione peroxidase (GPx) was observed in birds that fed on an AFT-contaminated diet when compared to other groups. Notably, AFT-triggered oxidative stress was substantially hampered by ChV supplementation exhibited by a dramatic reduction in the MDA level and drastic increases in GSH, SOD, and GPx indices.

### 3.5 Expression levels of pro-inflammatory genes in the kidney tissue

**FIGURE 3:**
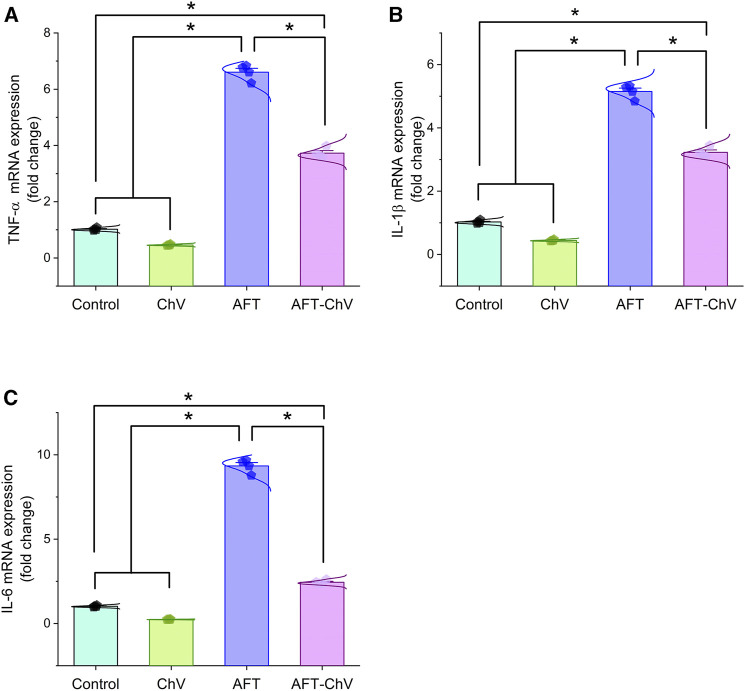
Bar-dot plot panel of mRNA expression of pro-inflammatory cytokines following ChV and/or AFT exposure in the kidney tissue. **(A)** TNF-α mRNA, **(B)** IL-1β mRNA, and **(C)** IL-6 mRNA. AFTs, aflatoxins; ChV, *Chlorella vulgaris*; IL-1β; interleukin-1β, IL-6, interleukin-6; TNF-α, tumor necrosis factor-α. Values are represented as mean ± SE (*p < 0.05).

As depicted in Figure 3, AFT could trigger renal inflammation indicated by considerable upregulation of mRNA expression levels of the pro-inflammatory cytokines (TNF-α, IL-1β, and IL-6) compared with controls. Nevertheless, birds that fed on a diet containing both ChV and AFT had a dampened inflammatory reaction in their kidneys, elucidated by the downregulation of the expression levels of the targeted pro-inflammatory genes.

### 3.6 Molecular docking

**FIGURE 4:**
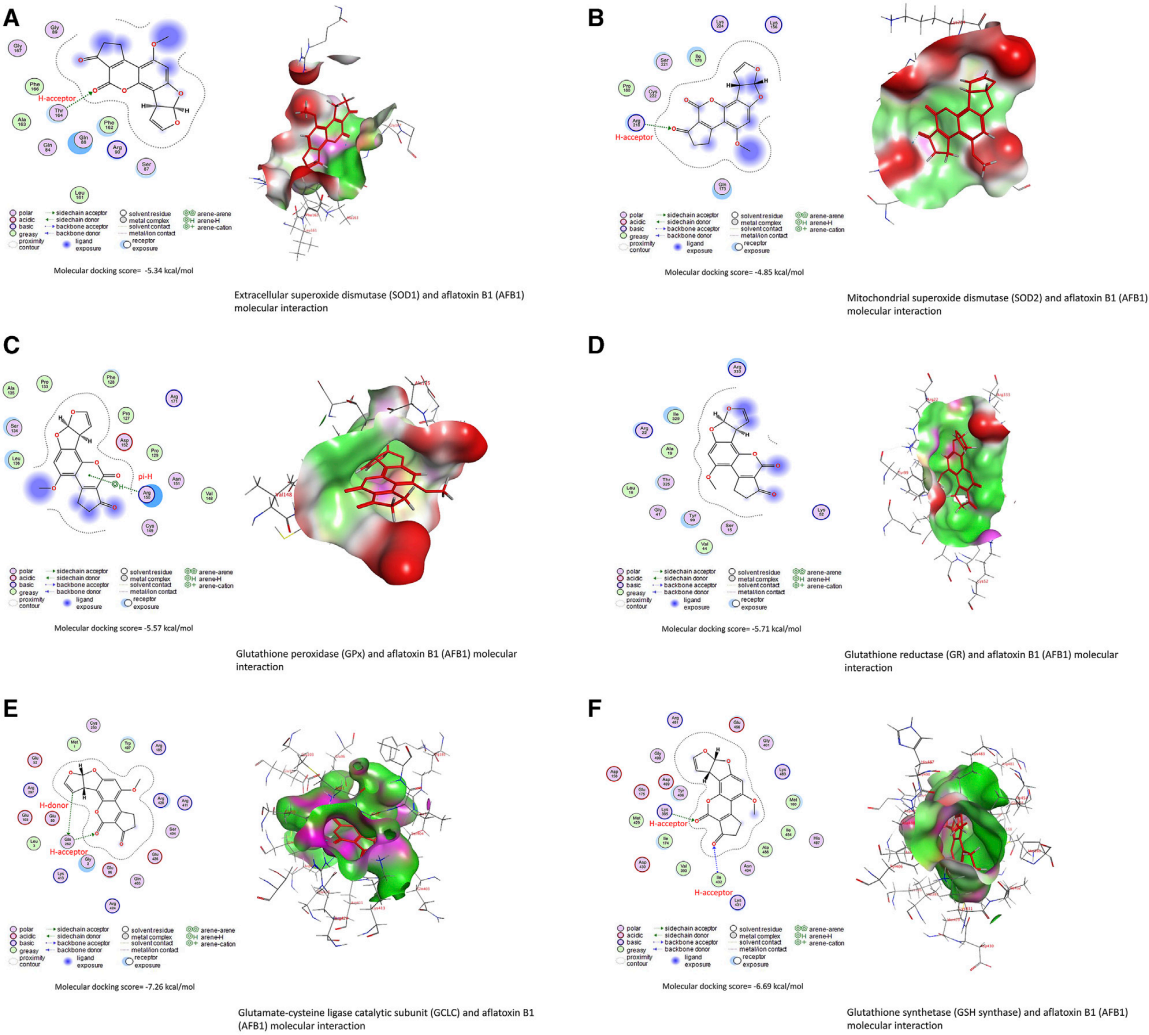
Molecular docking interactions of AFB1, AFB2, AFG1, and AFG2 with Japanese quails’ **(A)** extracellular superoxide dismutase (SOD1), **(B)** mitochondrial superoxide dismutase (SOD2), **(C)** glutathione peroxidase (GPx), **(D)** glutathione reductase (GR), **(E)** glutamate-cysteine ligase catalytic (GCLC) subunit, and **(F)** glutathione synthetase.

Data in Table 3 reveal the binding energy of AFT (B1, B2, G1, and G2) to the SOD1, SOD2, GPx, GR, GCLC, and GSH synthase binding sites of Japanese quails. AFB1 is the most abundant member of AFT in the naturally contaminated diet. The molecular docking dynamics revealed the great affinity of AFB1 to the SOD1, SOD2, GPx, GR, GCLC, and GSH synthase binding sites, with binding energy values of −5.34, −4.85, −5.57, −5.71, −7.26, and −6.69 kcal/mol, respectively (Figures 4A–F).

**TABLE 3:** Molecular docking scores of AFT on Japanese quails’ SOD1, SOD2, GPx, GR, GCLC, and GSH synthase.

AFT	Molecular docking score (kcal/mol)					
	SOD1	SOD2	GPx	GR	GCLC	GSH synthase
**AFB1**	−5.34	−4.85	−5.57	−5.71	−7.26	−6.69
**AFB2**	−5.15	−4.97	−5.58	−6.00	−7.30	−7.32
**AFG1**	−5.01	−5.20	−4.90	−5.66	−5.55	−7.17
**AFG2**	−5.18	−5.14	−5.71	−5.89	−5.78	−7.35

GCLC, glutamate-cysteine ligase catalytic subunit; GPx, glutathione peroxidase; GR, glutathione reductase; GSH synthase, glutathione synthetase; SOD1, extracellular superoxide dismutase; SOD2, mitochondrial superoxide dismutase.

**FIGURE 5:**
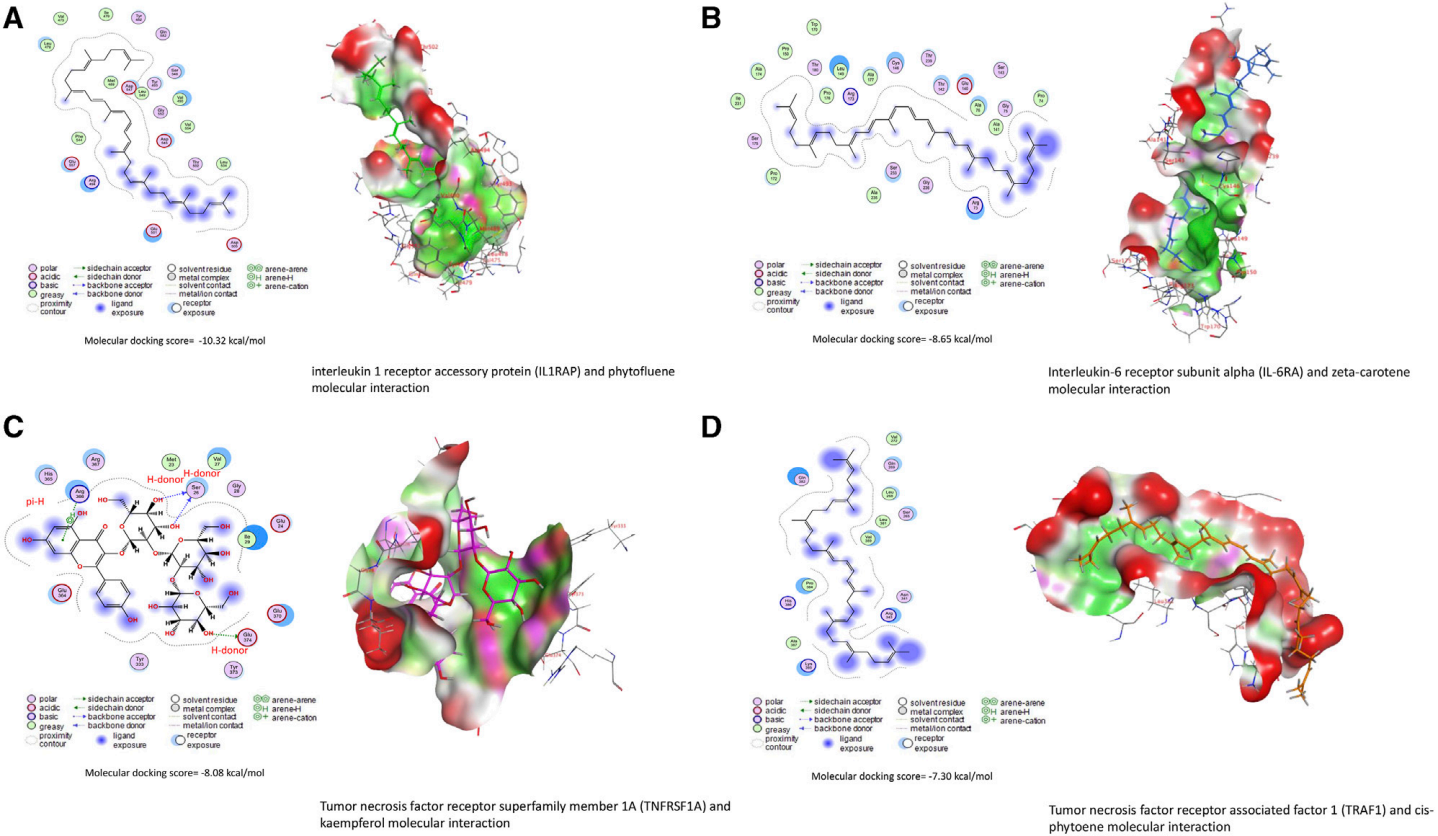
Molecular docking interactions of *Chlorella vulgaris* bioactive compounds with Japanese quails’ **(A)** interleukin-1 receptor accessory protein (IL1RAP), **(B)** interleukin-6 receptor subunit alpha (IL6RA), **(C)** tumor necrosis factor receptor superfamily member 1A (TNFRSF1A), and **(D)** tumor necrosis factor receptor-associated factor 1 (TRAF1).

Bioactive compounds of ChV interacted with the binding sites of IL1RAP, IL6RA, TNFRSF1A, and TRAF1, as shown in Table 4. The top five bioactive compounds, phytofluene, naringin, hesperidin, zeta-carotene, and *cis*-phytoene, bind to the binding site of IL1RAP, with binding energy values of −10.23 (Figure 5A), −10.21, −10.19, −9.74, and −9.71 kcal/mol, respectively. The binding site of IL6RA can interact with zeta-carotene (Figure 5B), rutin, phytofluene, kaempferol, and *cis*-phytoene, with binding energy values of −8.65, −8.48, −8.48, −8.46, and −8.46 kcal/mol, respectively. In addition, the binding site of TNFRSF1A can be targeted by kaempferol (Figure 5C), *cis*-phytoene, loroxanthin, rutin, and phytofluene, with binding energy values of −8.08, −7.80, −7.63, −7.55, and −7.36 kcal/mol, respectively. The binding site of TRAF can interact with *cis*-phytoene (Figure 5D), zeta-carotene, hesperidin, naringin, and rutin, with binding energy values of −7.30, −7.24, −7.11, −7.01, and −6.97 kcal/mol, respectively.

**TABLE 4:** Molecular docking scores of ChV’s bioactive compounds on Japanese quails’ IL1RAP, IL6RA, TNFRSF1A, and TRAF1.

Compound	Molecular docking score (kcal/mol)			
	IL1RAP	IL6RA	TNFRSF1A	TRAF1
7-Hydroxyflavanone	−6.28	−5.47	−4.96	−4.76
Benzoic acid	−4.79	−4.49	−4.16	−3.97
Caffeic acid	−5.40	−4.91	−4.41	−4.59
Catechin	−6.95	−5.60	−4.96	−5.53
Catechol	−5.00	−4.28	−4.02	−3.66
Chlorogenic acid	−8.19	−6.29	−6.14	−5.90
Cinnamic acid	−5.11	−4.73	−4.13	−4.23
*cis*-Phytoene	−9.71	−8.46	−7.80	−7.30
Ellagic acid	−6.70	−5.78	−5.11	−5.04
Gallic acid	−5.44	−4.92	−4.49	−4.08
Hesperidin	−10.19	−7.67	−7.14	−7.11
Kaempferol	−8.52	−8.46	−8.08	−6.77
Loroxanthin	−8.90	−7.91	−7.63	−6.33
Luteolin	−6.82	−5.86	−5.07	−5.01
Naringin	−10.21	−8.43	−7.10	−7.01
Phytofluene	−10.23	−8.48	−7.36	−6.69
Pyrogallol	−5.12	−4.22	−3.97	−3.82
Quercetin	−7.00	−6.16	−4.89	−5.40
Rutin	−9.17	−8.48	−7.55	−6.97
Salicylic acid	−5.46	−4.64	−4.05	−3.96
Syringic acid	−5.73	−5.10	−4.68	−4.66
Zeta-Carotene	−9.74	−8.65	−7.35	−7.24
*β*-Carotene	−9.23	−7.77	−7.01	−6.57

IL1RAP, interleukin-1 receptor accessory protein; IL6RA, interleukin-6 receptor subunit alpha; TNFRSF1A, tumor necrosis factor receptor superfamily member 1A; TRAF1, tumor necrosis factor receptor-associated factor 1.

### 3.7 Assessment of AFT residues in eggs

**FIGURE 6:**
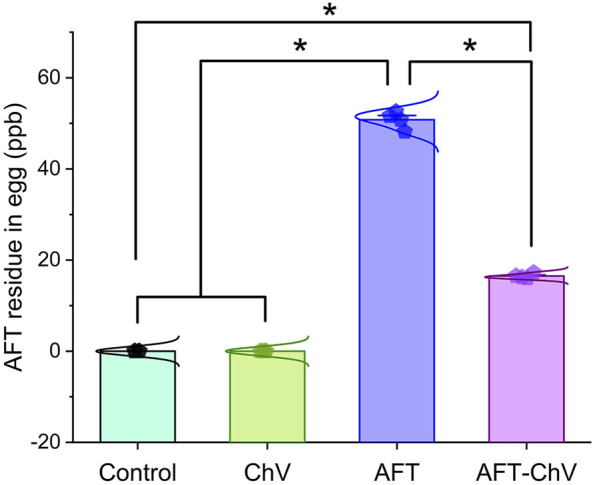
Bar-dot plot panel of the impact of ChV supplementation on egg total AFT residue. AFTs, aflatoxins; ChV, *Chlorella vulgaris*. Values are represented as mean ± SE (*p < 0.05).

Figure 6 shows that birds that fed on the AFT-contaminated diet displayed a noticeable increase in the level of AFT residues in their eggs compared with birds that fed on a basal diet or supplemented with ChV solely. However, supplementation with ChV could lessen the accumulation of AFT in the eggs of birds that fed on an AFT-contaminated diet.

### 3.8 Biological networks, hierarchical clustering heatmap, and variable importance in projection (VIP) score

**FIGURE 7:**
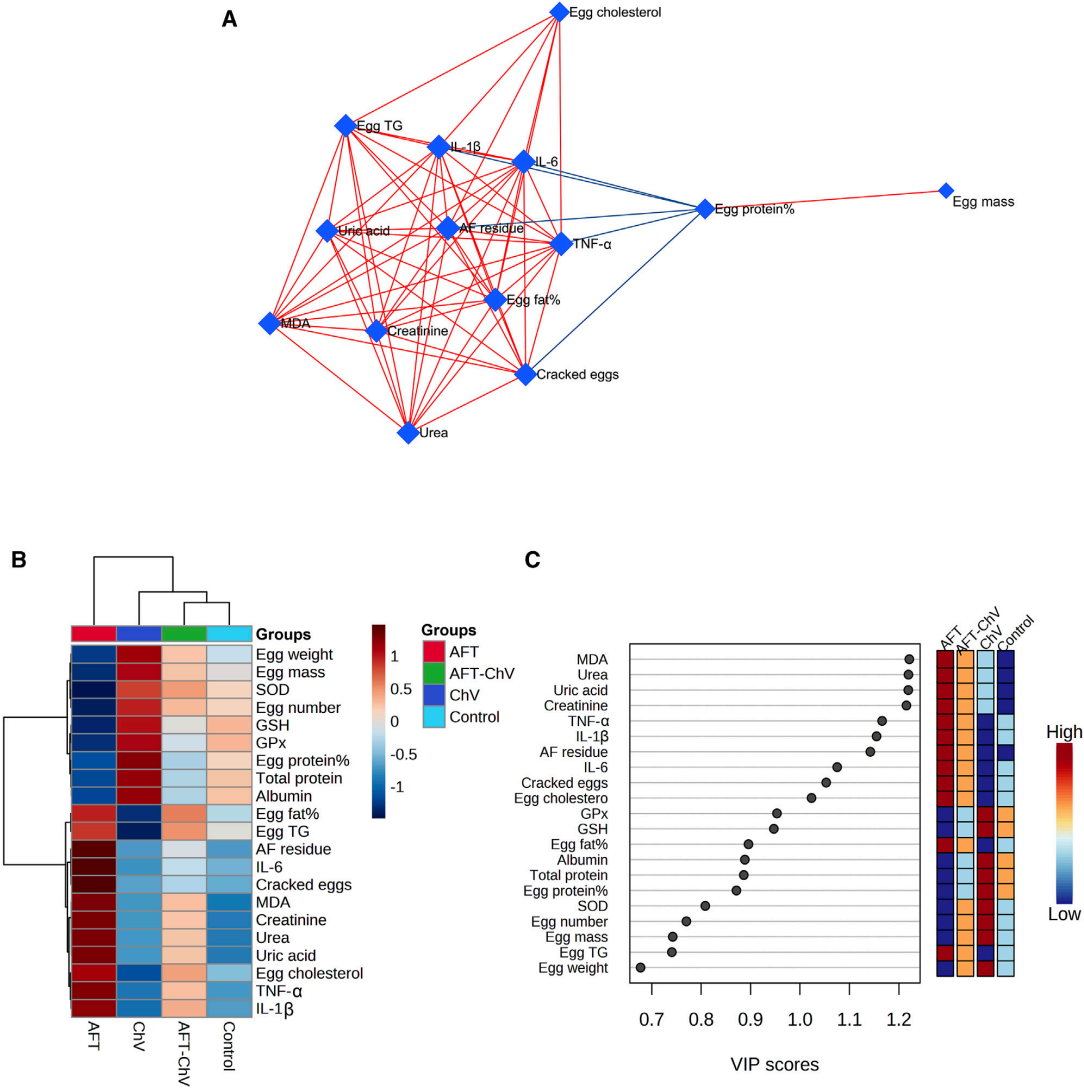
Clustering analysis of whole datasets after ChV and/or AFT exposure. **(A)** DSPC network of substantially distinct variables in the control and exposed groups. In the DSPC network, the nodes represent the measured variables, while the edges signify the correlation measures. Variables with the stronger correlation group cluster together and have wider edges between them. The blue lines display a negative correlation, while the red lines display a positive correlation with variables. **(B)** Heatmap and hierarchical clustering provide a visual summary of all the data. Each colored cell on the map represents a concentration value, and the rows and columns are made of different averages and treatment sets, respectively. Dark red has the highest value on the gradation scale, while blue has the lowest. **(C)** VIP score; the average concentrations of the measured variables are displayed for each study group in colored boxes on the right, and a colored scale from maximum (red) to least (blue) represents the contribution strength. AFTs, aflatoxins; ChV, *Chlorella vulgaris*; DSPC, debiased sparse partial correlation; VIP score, variable importance in projection score.

Multivariate analyses were conducted to determine the association between various parameters and treated groups, as shown in Figure 7. Biological networks of all variables were constructed. The nodes display various parameters, while the lines depict the relationships among these variables in the DSPC algorithm network (Figure 7A). During the data normalization stage, the data were changed to log or cubic roots for the best performance. The DSPC network is more suitable for creating biologically pertinent networks and discovering unidentified substances. The variable that is closer to the center exhibits a stronger association with these selected parameters and the most relevant position in the network, such as AFT residue. On the other hand, AFT residue was positively correlated with the majority of examined parameters while negatively correlated with egg protein%. Furthermore, the metabolic pathway network was also constructed to explore the relationships between the most disrupted pathways and various parameters induced by AFT exposure.

The clustering heatmap exemplifies an evident visual depiction of all datasets (Figure 7B) and reveals a notable discrepancy in the concentration of whole measured parameters in response to AFT toxicity compared to other groups. These findings posit that the quails that fed on an AFT-contaminated diet exhibited greater damage than those of other groups.

Additionally, according to the VIP score, the top influential factors in our study were MDA, urea, uric acid, creatinine, TNF-α, IL-1β, and AFT residue which were sensitive to various treatments and could distinguish AFT treatment from others (Figure 7C).

## 4 Discussion

AFT is the most hazardous toxin that frequently contaminates grains, food, and feedstuff and remains after food processing, raising serious health concerns (Wang et al., 2022). As its metabolites (AFTO) preferentially accumulate in the renal tissue during urinary excretion, it has been postulated in a number of studies as a potential contributor to renal disorders (Yilmaz et al., 2018; Abdel-Daim et al., 2021).

Ample literature, including our and others’ preceding studies, strongly indicate that excess oxidant production and cellular antioxidant depletion are fundamental pathways embroiled in AFT-induced nephrotoxicity (Owumi et al., 2020; Abdel-Daim et al., 2021; Wang et al., 2022). This, owing to the ability of AFT to directly attack the cellular macromolecules, notably, nucleic acid and proteins, results in immense ROS formation, including superoxide anions (O_2_^•–^), hydroxyl radicals (OH^•^), and hydrogen peroxide (H_2_O_2_). Interestingly, SOD is an antioxidant enzyme that promotes the breakdown of O_2_^•−^ radical into O_2_ and H_2_O_2_, thereby annihilating oxidative damage (Wispriyono et al., 2021). Additionally, GSH plays a crucial role as a co-substrate for enzyme GPx, which is in charge of detoxifying the H_2_O_2_ and lipid hydroperoxides, as well as direct neutralization of AFT (by forming an AFT–GSH conjugation) (Kemal and Seker, 2023). However, Fenton’s reaction is initiated when antioxidants are depleted by AFT-initiated ROS; therefore, enormous amounts of OH^•^ are built up, which aggressively attack the phospholipid membrane, leading to MDA accumulation (Aboubakr et al., 2021; Abdelnaby et al., 2022). MDA is the byproduct of lipid peroxidation (LPO) that represents the degree of membrane damage. It could be more detrimental to human health than AFT itself through engaging with essential intracellular molecules, thus expediting oxidative injury (Fouad et al., 2019).

Accordingly, oxidative damage is prominently emphasized in our study, expounded by an evident drop in GSH levels and the activities of SOD and GPx in renal tissues. The ongoing work has also further proven the detrimental impact of AFT-induced LPO on the tubular epithelial membrane expounded by the substantial elevation of the antioxidant enzymes such as MDA. As a consequence, tubular dysfunction occurs and is demonstrated by a significant upsurge in the serum levels of urea, creatinine, and uric acid. These results support our preceding research, which revealed a positive association between the MDA level and the increased serum levels of renal function parameters (Abdelnaby et al., 2022). Our findings are in agreement with those of Manafi (2018) who reported that the albumin level is negatively affected when Japanese quails are treated with AFT. Furthermore, as described in previous reports, the current study revealed evident decreases in serum albumin and total protein levels following AFT exposure. Such decreases might be attributed to impaired tubular reabsorption with increased urinary protein loss (Abdeen et al., 2020). In other words, the accumulated MDA may trigger further DNA and protein oxidation, resulting in the inhibition of the processes of translation and transcription of mRNA and thence protein synthesis (Abdeen et al., 2021; Aboubakr et al., 2021).

Furthermore, the elevated levels of ROS due to AFT exposure led to the deletion of antioxidant status in the renal tissue, as evidenced by significant reductions in GSH, SOD, and GPx in the current study. In addition, the molecular docking study revealed the binding interaction between AFT and SOD1, SOD2, GPx, GR, GCLC, and GSH synthase. In the same context, AFT significantly elevated MDA and reduced whole-blood GSH (Citil et al., 2005) and hepatic SOD and GPx (Elbasuni et al., 2022) in Japanese quails.

In conformity to accumulating evidence, inflammation and oxidative stress are remarkably correlated. Increased ROS production boosts the nuclear factor kappa B (NF-κB) signaling cascade and promotes the expression of pro-inflammatory cytokine genes, resulting in a profound inflammatory reaction (Ahmed et al., 2022). TNF-α is the most important pro-inflammatory cytokine that is implicated in the activation of NF-κB, stimulating the regulation of interleukins and other inflammatory mediators downstream (Rajendran et al., 2018). Our results support the aforementioned mechanism, exhibited by enhanced gene expression of inflammatory factors, TNF-α, IL-1β, and IL-6, in response to AFT intoxication. Thus, we propose that the inflammatory process is a possible pathway that involved AFT-induced nephropathy. These findings are consistent with those of Gao et al. (2021) who observed the overexpression of these inflammatory cytokines in chick kidneys upon AFT exposure.

Regarding the effect of AFT on the nutritional value of eggs, contrary to other treated groups, AFT had an impact on the nutritional value of eggs collected from quails subjected to AFT. That was exhibited in the current work by reduced protein content and elevated levels of total fat, cholesterol, and triacylglycerol contents. Since the ROS produced by AFT substantially induces DNA adducts and protein oxidation, protein synthesis and lipid metabolism are negatively affected (Abdel-Daim et al., 2020; 2021; Ahmed et al., 2022). As anticipated, the current trial proved the presence of substantial residuals of AFT in eggs, which has an impact on the safety and quality of edible components of birds that fed on an AFT-contaminated diet. Manafi (2018) reported that AFT metabolites are carried over from quail feed to eggs and thence to the consumer. This finding agrees with that of Oliveira et al. (2003) who identified AFT residues in the eggs of laying quails.

A plethora of research has investigated the economic implications of various levels of AFT in egg production. A reduction in egg number along with egg weight was observed by Oliveira et al. (2003) following dietary AFT inclusion in Japanese quails, similar to that reported in the current work. This reduction in egg production and quality may be due to the negative impact of AFT on egg development through the disruption mobilization of fat from the liver to the ovary (Manafi, 2018). Moreover, our data demonstrated poor eggshell quality in quails that fed on an AFT-contaminated diet, and this is due to reduced deposition of calcium in the bone after AFT exposure, which accounts for one-third of the calcium supply required for eggshell formation. AFT-induced malfunction of the kidney leads to decreased synthesis and activation of vitamin D and thus decreased calcium deposition in the bone (Fouad et al., 2019).

ChV is a green microalga frequently used as a prophylactic remedy to maintain renal health due to its renowned antioxidant, anti-inflammatory, and immune-modulating properties (Blas-Valdivia et al., 2011; Al-Halaseh et al., 2022). The antioxidant capability has been related to its phenolic constituents identified among other active phytoconstituents such as carotenoids, lutein, catechin, carotenoids, gallic acid, caffeic acids, benzoic acid, chlorogenic acid, and rutin (Blas-Valdivia et al., 2011; Andrade et al., 2018; Prabakaran et al., 2019; Sikiru et al., 2019). Additionally, it contains a variety of essential trace elements (Cu, Zn, Se, and Fe) that are required for the function of numerous antioxidant metalloenzymes (Abu-Serie et al., 2018).

Accumulating evidence corroborates that ChV consumption counteracts oxidative stress via modulating antioxidant enzymes, scavenging the free radicals, and mitigating LPO (Sikiru et al., 2019). This hypothesis is supported by our finding, indicated by improvements in renal function and oxidant/antioxidant status in birds concurrently treated with AFT and ChV. Our findings agree with the previous reports that ChV supplementation drastically decreased the generation of MDA along with increased antioxidant enzyme levels in a fish model (Abdelhamid et al., 2020). Another report documented increased antioxidative enzyme activities in rabbits fed on ChV supplements (Abdelnour et al., 2019a).

Along with its antioxidant activity, ChV exhibits a well-known potent anti-inflammatory property via regulating inflammatory cytokine release, including IL-6, TNF-α, and iNOS, as well as inhibiting proteinase and lipoxygenase activities, which are implicated in the inflammatory process (Prabakaran et al., 2019; Abdelhamid et al., 2020). Additionally, the molecular docking study revealed the powerful binding of ChV bioactive compounds to the binding sites of IL1RAP, IL6RA, TNFRSF1A, and TRAF1 in quails. Accumulating evidence indicates that ChV’s potential to mitigate inflammation is ascribed to its abundance in PUFA, which plays a crucial role in regulating the release of pro-inflammatory cytokines and relieving cellular inflammation (Abdelnour et al., 2019a; Remize et al., 2021; Yang et al., 2022). This is in agreement with Abdelhamid et al. (2020) who reported that ChV supplementation contributed to the downregulation of TNF-α in splenic fish. Another report elucidated that ChV’s ability to suppress inflammation might be attributed to its capacity to upmodulate cellular antioxidant indices while downregulating inflammatory mediators (Abu-Serie et al., 2018). In an endorsement of previous studies, the current study elucidates the anti-inflammatory action of ChV, as explained by the downregulation of inflammatory cytokines.

It is worth mentioning that the findings of our research highlight the economic benefits of ChV as an egg production enhancer and nutritional value promoter when compared to the AFT group. ChV contains a plethora of high-quality nutrients, such as vital amino acids, vitamins, and minerals, and functions as a growth enhancer and productive booster (Lamminen et al., 2019). Additionally, supplementation of ChV reduced the AFT residue compared to the AFT group in our trial. Such enhancement might be related to ChV’s antioxidant activity, improving renal function and, in turn, the metabolic processes. According to Zheng et al. (2012), feeding diets supplemented with ChV enhanced the eggs’ morphological traits, which concurs with the current study. Furthermore, the results indicate that ChV could enhance egg quality and laying performance of laying hens, which is in agreement with previous studies (Halle et al., 2009).

**FIGURE 8:**
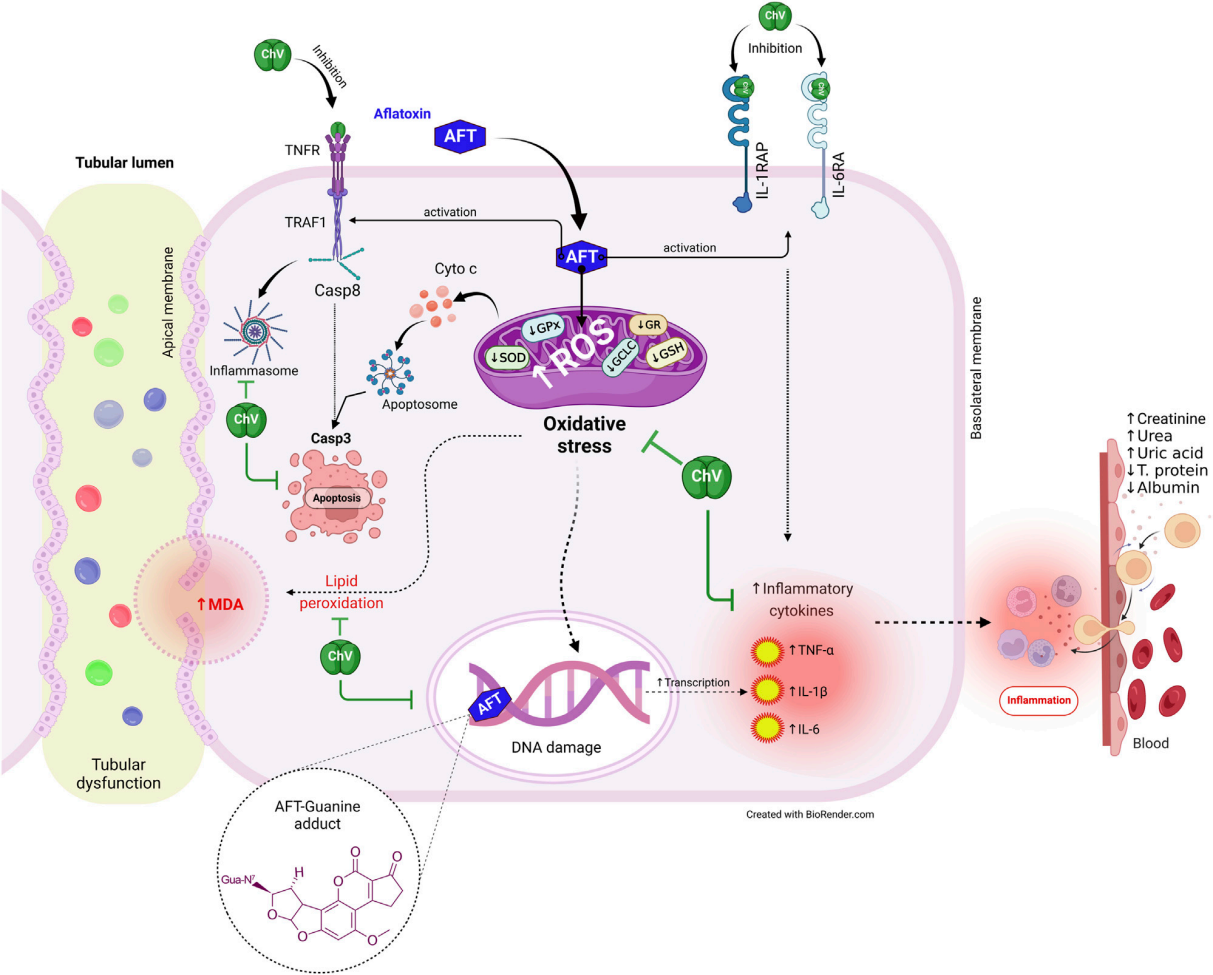
The protective effect of ChV against AFT-induced kidney injury is underpinned by molecular processes. AFTs, aflatoxins; ChV, *Chlorella vulgaris*; GCLC, glutamate-cysteine ligase catalytic subunit; GPx, glutathione peroxidase; GR, glutathione reductase; GSH synthetase, glutathione synthetase; IL1RAP, interleukin-1 receptor accessory protein; IL6RA, interleukin-6 receptor subunit alpha; MDA, malondialdehyde; ROS, reactive oxygen species; SOD, superoxide dismutase; TNFR, tumor necrosis factor receptor; TRAF1, tumor necrosis factor receptor-associated factor 1.

Additionally, multivariate statistical analyses using the DSPC network, clustering heatmap, and VIP score were carried out to analyze the variable contributions affected by different treatments on renal tissue and quail eggs. The DSPC network revealed that AFT residue exhibits the more relevant position in the network and positively correlated with the majority of examined parameters while negatively correlated with egg protein%. The clustering heatmap effectively epitomizes that AFT exposure induced significant alterations in all examined parameters compared to other treatment groups, indicating prospective improvements in those parameters when ChV was supplemented. MDA, urea, uric acid, creatinine, TNF-α, IL-1β, and AFT residue were also found to be the top impacting variables in our study according to the VIP score. The molecular pathways underpinning ChV’s potential to protect against kidney damage induced by AFT are shown in Figure 8.

## 5 Conclusion

AFT induces renal dysfunction via the stimulation of oxidative stress, LPO, and inflammatory response, which leads to decreased egg nutritional value and increased AFT accumulation in the egg. ChV supplement has the capability to safeguard the kidney from the detrimental effects of AFT. This is likely due to ChV’s enriched nutritional constituents and antioxidant, ROS-scavenging, and anti-inflammatory attributes. The antioxidant, inflammatory, and apoptotic proteins targeted by ChV active constituents were validated by molecular docking dynamics. Thus, to mitigate the potentially harmful effects of AFT on both humans and animals, we strongly suggest that ChV supplementation may be a promising, secure, and economical biological approach.

## Data Availability

The original contributions presented in the study are included in the article/Supplementary Material; further inquiries can be directed to the corresponding authors.
