# Supplementary material for: Microalgae (Chlorella vulgaris) attenuates aflatoxin-associated renal injury
Source: Front Pharmacol. 2023 Dec 27;14:1291965. doi: 10.3389/fphar.2023.1291965 (PMC10777483; doi:10.3389/fphar.2023.1291965)
Supplement: Supplementary file 1 [file Table1.DOCX]

**Table S1.** Forward and reverse primer sequences used for qRT-PCR

| **Gene** | **Primer sequence** | **Reference** |
| --- | --- | --- |
| 28S rRNA | F: GGCGAAGCCAGAGGAAACT  R: GACGACCGATTTGCACGTC | [31] |
| TNF-α | F: CCCCTACCCTGTCCCACAA  R: ACTGCGGAGGGTTCATTCC | [31] |
| IL-1β | F: GCTCTACATGTCGTGTGTGATGAG  R: TGTCGATGTCCCGCATGA | [31] |
| IL-6 | F: GCTCGCCGGCTTCGA  R: GGTAGGTCTGAAAGGCGAACAG | [31] |
